# The Parallel Mediation Effects of Depression, Well-Being, and Social Activity on Physical Performance and Frailty in Community-Dwelling Middle-Aged and Older People

**DOI:** 10.1155/2022/7979006

**Published:** 2022-12-12

**Authors:** Eva Berthy Tallutondok, Chia-Jung Hsieh, Pei-Shan Li

**Affiliations:** ^1^School of Nursing, College of Nursing, National Taipei University of Nursing and Health Sciences, Taipei, Taiwan; ^2^Faculty of Nursing, Universitas Pelita Harapan, Tangerang, Banten 15811, Indonesia; ^3^Department of Nursing, Taipei Veterans General Hospital, Taipei 112303, Taiwan

## Abstract

**Background:**

Frailty refers to a decline in an elderly person's physical, psychological, and social functioning, making them sensitive to stressors. Because frailty is caused by a variety of factors, including certain demographic characteristics, understanding the mediating factors that affect frailty in the elderly is critical.

**Purpose:**

To provide evidence about the relationship between depression, well-being, social activity, physical performance, and frailty among older adults.

**Materials and Methods:**

The study used secondary data from Taiwan's Long-term Study of Aging (*n* = 7,622), excluding people with severe dementia. The chi-square test and Spearmen's coefficient correlation were used to assess the relationship between the demographic variables and frailty. Nonparametric bootstrapping analysis was used to test whether depression, well-being, and social activity are parallel mediators of the relationship between physical performance and frailty. This study was approved by Fu Jen Catholic University (FJU-IRB No. C110040).

**Results:**

The overall frailty prevalence was 13.9%. We calculated a mean score and standard deviation for each measurement in this study. The correlation found low-to-moderate positive and negative statistically significant correlations between the variables. A significant, moderately negative relationship was found between physical performance and frailty that correlated with three potential mediating factors. The path indicated that lower physical performance scores and higher depression scores are more likely to be associated with frailty.

**Conclusion:**

Older adults who are depressed are more likely to become frail. Adults who are more socially active and report greater well-being are less likely to become frail. Therefore, further research should design and test a comprehensive intervention for older adults in community settings that addresses all three factors, aimed at increasing well-being and social activity while also treating depression.

## 1. Introduction

Meeting the particular needs of frail older adults in community settings would improve health outcomes and quality of life while also lowering health care costs in any country [1, 2]. Frailty refers to a multidimensional loss of human functioning that may be physical, psychological, or social [3]. Moreover, frailty increases as people age, from a prevalence of 30% among people aged 60 and older to 40% among those over 70, so it presents a burden for individuals and families in the community [4]. The presence of frailty, poor health, and multiple comorbidities results in a high risk of adverse outcomes, such as falls and disability [5]. Therefore, identifying frailty is important in providing clinical care for older adult community-dwelling populations.

Previous studies have shown that demographic factors contribute to frailty, such as being female and increasing age [6, 7], while medical factors also contribute to frailty, such as cognitive impairment, sarcopenia, falls, and institutionalization [8–12]. However, mediation studies have revealed that other factors associated with frailty also include physical condition, psychological status, and social activity.

The literature documents associations between frailty and these three additional variables. For example, a study that examined the relationship between frailty and physical performance (i.e., movement, behavior, and body composition) found that it was mediated by a person's physical activity, with sedentary time and moderate-to-vigorous physical activity acting independently as mediators [13]. A study of the association between frailty and cognitive function found that it was mediated by psychological distress; those suffering higher levels of psychological distress had higher rates of frailty [14]. Another study found that the association between frailty and loneliness was mediated by social activity and engagement [15]. However, a cross-sectional study of the relationship between frailty and social activity among older adults in Japan during the COVID 19 pandemic found no link between frailty and being hindered in social activity [16]. This finding might be explained by the unique conditions of the pandemic, which limited social activity for frail and not-so-frail people alike [16].

The presence of comorbidities is an understudied predictor of frailty, so the evidence is limited. Studies of the relationship between frailty and physical disease in community-dwelling older adults have mostly used simple and serial mediation; few studies have used parallel mediation, which can include more than two variables [17]. However, parallel mediation analysis has yielded interesting results in identifying symptoms related to frailty [17]. The principles of parallel mediation used in this study are described in the work of Kane and Ashbaugh in 2017 and Hayes and Preacher in 2014 [18, 19]. We evaluated the magnitude of the degree correlation according to the work of Hopkins [20], namely, robust (*r* = 0.7-0.8), strong (*r* = 0.5–0.7), moderate (*r* = 0.3-0.4), small (0.1-0.2), and trivial (*r* < 0.1) This study used parallel mediation analysis to evaluate the effects of depression, well-being, and social activity on the relationship between frailty and physical performance.

## 2. Materials and Methods

### 2.1. Study Design and Sample

This study used secondary data from a cross-sectional study, the 2015 Taiwan Longitudinal Study on Aging (TLSA). The participants were thus not directly involved in this study. The total TLSA sample data consisted of 8,300 older adults in Taiwan who were divided into two age groups, those aged 50 to 64 and those aged 65 to 85. After excluding older adults with severe dementia, the total number of participants in the study was 7,622. Participants' data were anonymized to protect privacy and human rights according to the Declaration of Helsinki guidelines on research with human subjects. This study was approved by Fu Jen Catholic University (FJU-IRB No. C110040).

### 2.2. Measurement

#### 2.2.1. The Instrumental Activities of Daily Living (IADL) Scale

The IADL scale, created by Lawton and Brody, has been used to measure physical performance since 1969 and consists of nine items representing independent living skills. The Chinese version of the IADL has a Cronbach's alpha range of 0.82 to 0.92 [21]. The scale measures difficulty performing nine tasks, each with two possible responses, yes (able) or no (not able). “Yes” answers are given one point, and “no” answers are assigned a value of zero; the total scores thus range from zero to nine. The TLSA and this study used the IADL to measure physical performance as an independent variable.

#### 2.2.2. The Geriatric Depression Scale-15 (GDS-15)

The GDS-15 short form is used to measure depression; it was introduced by Yesavage in 1982. It includes fifteen items with two possible responses: yes or no. Depression levels are categorized as follows: scores from 0 to 4 indicate normal; scores from 5 to 8 indicate mild depression; scores from 9 to 11 indicate moderate depression; and scores from 12 to 15 indicate severe depression. For 10 of the items, positive responses are assigned 1 point, and for 5 items, negative responses are assigned 1 point (items 1, 5, 7, 11, and 13). The Cronbach's alpha coefficient for the total scale is 0.80 [22].

#### 2.2.3. World Health Organization-5 (WHO-5) Scale

The WHO-5 scale is used to evaluate well-being in older adults. It consists of 5 items; each item can have a score from 0 to 5, with the overall raw score ranging from 0 to 25. All scores are then multiplied by 4, with the highest total score being 100. Cronbach's alpha coefficient for this scale ranged from 0.81 to 0.86 [23].

#### 2.2.4. The Social Activity Scale (SAS)

The SAS was developed in Japan to measure social activity among older adults in community settings [24]. Items asked about activities at home or in the community, with responses “yes” and “no” assigned point values of one and zero, respectively. The highest possible score for social activity is 15, which indicates having leisure time and social life in the community. This 15-item scale has Cronbach's alpha of 0.791 [24].

#### 2.2.5. Frailty

This study measured frailty by evaluating the physical, psychological, and social conditions of older adults in community settings using the Tilburg Frailty Indicator [3]. It has a total possible score of 15, with scores of 5 or more indicating frailty [3]. For this study, the variables representing frailty were constructed from the TLSA 2015 data [25, 26].

Control variables were demographic characteristics such as age, gender, education, marital status, and economic condition, as well as diagnoses in the past year and the number of chronic diseases.

### 2.3. Statistical Analysis

Analysis was performed using the process function of SPSS version 22.0 statistical software. In this study, 9%–12% of participants had missing data on demographic characteristics and were excluded from this study. The continuous variables are displayed using means and standard deviations (SDs), and the categorical variables are displayed using case numbers and percentages (%). The chi-square test was used to assess the relationship between frailty and the demographic variables, which were then assessed for significance using the crosstab function of SPSS. Mentioning the *p* values in the demographic characteristics is that *p* value is important for developing a rigorous statistical analysis to consider whether any variables in the demographic data could potentially affect the study's results. Data were examined to assess the normality of the distribution using the Shapiro–Wilk test, a histogram, and Q-Q plots. Since the data were not normally distributed, nonparametric, and dichotomous, Spearman's correlation coefficient was used to investigate the statistical relationship between the variables [27]. Spearman's correlation analysis was conducted to determine the relationships among five variables, physical performance, depression, well-being, physical activity, and frailty, all of which were statistically significant with *p* values less than 0.05.

Nonparametric bootstrapping analysis [19] was used to test depression, well-being, and social activity as potential parallel mediators of the relationship between frailty and physical performance. The mediation is significant if the CI is 95% and the bias-corrected lower and upper limits for indirect effects do not include zero [19]. Three mediation models were developed to test the hypothesis that depression, social activity, and well-being affect the relationship between physical performance and frailty. Parallel mediation effects were tested using model four of the bootstrap method of Preacher and Hayes. We used the PROCESS function version 4.0 in SPSS version 22 to analyze mediation.

## 3. Results and Discussion

### 3.1. Results

#### 3.1.1. Demographic Characteristics

Descriptive demographic characteristics of the participants (*n* = 7,622) are shown in Table 1. The prevalence of frailty was 13.9%, and 86.1% of older adults were robust. A total of 87.78% of the participants had no chronic diseases; 82.71% of the participants had a high school education or below; and 78.75% of the participants were satisfied with their economic status. Moreover, the results showed statistically significant correlations between frailty and gender, age, education, marital status, having a disease diagnosed in the past year and chronic diseases. The exception was the association between frailty and satisfaction with economic status, which was not statistically significant (*p* = 0.196).

#### 3.1.2. Correlations between Frailty and Physical Performance, Depression, Well-Being, and Social Activity

Spearman's correlation shows the relationships among physical performance, depression, well-being, social activity, and frailty (Table 2). We calculated mean scores and standard deviations for each of these variables. Spearman's correlation found both positive and negative, statistically significant (*p* < 0.01), low-to-moderate relationships with frailty. Physical performance had a significant negative association with depression (*rho* = −0.245) and frailty (*rho* = −0.562), but it was positively associated with well-being (*rho* = 0.216) and social activity (*rho* = −0.335; *p* < .01).

#### 3.1.3. Testing the Partial Mediation Models

Partial mediation analysis found that both direct and indirect mediating effects were significant (*p* < 0.001), with 95% confidence intervals in bootstrap analysis (Table 3). Model 1 showed that depression negatively mediated the relationship between physical performance and frailty. Models 2 and 3 tested positive for mediation. The effect of depression on frailty was positive (*β* = 0.20; *p* < 0.001), but well-being (*β* = −0.19; *p* < 0.001) and social activity (*β* = −0.18; *p* < 0.001) had no effect on frailty.

#### 3.1.4. Testing Parallel Mediation Models

**Figure 1:**
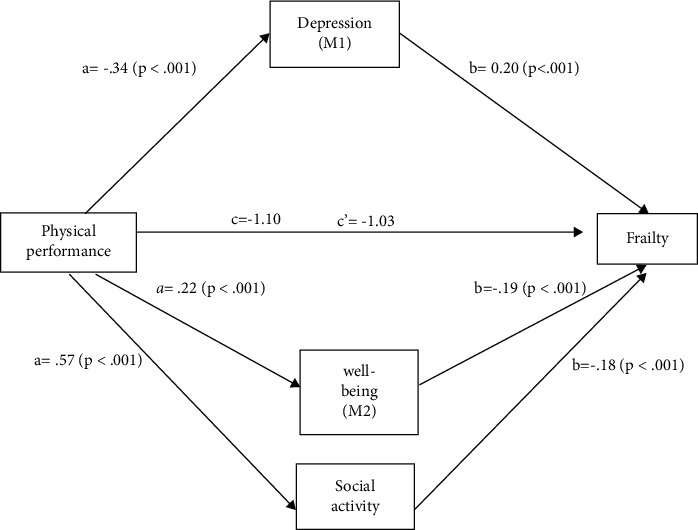
Parallel mediation models. Direct effect of physical performance and frailty is *β* = −1.03 (SE = 0.01). Total effect of physical performance and frailty is *β* = −1.10 (SE = 0.01). Total indirect effects of BootLLCI∼BootULCI are none containing zero: depression (*β* = −0.05, 95% CI: (−0.05∼−0.04), *p* < 0.001); well-being (*β* = −0.03, 95% CI: (−0.03∼−0.02), *p* < 0.001); social activity (*β* = 0.07, 95% CI: (−0.07∼−0.06), *p* < 0.001).

In the path, the direct effect of (a) physical performance on depression was negative and statistically significant (*β* = −0.34; *p* < 0.001), (b) the direct effect of depression on frailty was positive and significant (*β* = 0.20; *p* < 0.001), while (c) the direct effect of physical performance on frailty is negative and statistically significant (*β* = −1.03; *p* < 0.001) (Figure 1). The path indicates that older adults who have lower scores on physical performance and higher scores on depression are more likely to be frail.

### 3.2. Discussion

#### 3.2.1. Demographic Characteristics

This article investigated the potential mediating factors in the relationship between physical performance and frailty. The results indicated that the prevalence of frailty was 13.9%. More than three-quarters of the participants had no chronic disease, and a similar proportion had lower education levels. However, both chronic disease and education were significantly correlated with frailty in this study. This finding is consistent with previous studies in Taiwan that also found that level of education and chronic disease were significantly correlated with frailty [26]. Other evidence indicates that a lack of education is linked to poor cognitive performance, functional disability, and weakness, all characteristics of frailty [28–30]. Another study found that patients who become frail due to chronic diseases did not differ from the other groups in terms of functional, cognitive, or psychological status. Older adults with chronic diseases had a higher comorbidity burden and more frailty [31]. Therefore, in order to get clear data about frailty, patients with dementia were excluded from this study.

#### 3.2.2. Association between Study Variables

This study demonstrated associations between frailty and physical performance, depression, well-being, and social activity (Table 2). The association between physical performance and depression was negative and significant, as was the relation between physical performance and frailty. These findings are consistent with previous studies, which found that physical performance was negatively associated with depression scores [13, 29, 32]. Other studies have found four functional limitations associated with frailty: (1) physical functioning, (2) activities of daily living, (3) instrumental activities of daily living (IADL), and (4) constraints on social participation [33]. Weak upper- and lower-body physical performance was associated with depression among older adult women [34]. The results of this and other studies indicate that physical impairments are crucial predictors of depression and frailty in Taiwan. In turn, depression might be a mediating factor in the relationship between physical performance and frailty.

In contrast, the association between physical performance and well-being was significantly positive but low, while well-being and frailty had a low negative correlation. Previous studies found that physical activity has a positive effect on well-being among older adults [35]. For example, social media used as an IADL significantly predicts social well-being among older adults in the twenty-first century [36]. Others have shown that aspects of well-being, such as a sense of autonomy, control, purpose, and fulfillment, as well as happiness and pleasure, protect against the development of frailty [37]. In addition, cultural differences in how people define well-being may be explained by differences in norms about appropriate emotions and the importance of mental health [38]. Moreover, the correlation between physical performance and well-being may differ by region or country [39]. Furthermore, unique individual differences may impact these relationships; for example, maladaptive psychological responses would negatively impact psychological well-being, which might then contribute to frailty [40]. In this study, well-being was found to be a mediating factor in the relationship between physical performance and frailty; therefore, we should pay attention to the mental well-being of older adults in order to reduce the risk of frailty.

In this study, social activity was found to be a mediating factor in the relationship between physical performance and frailty. Our results showed a low but significant positive correlation between social activity and physical performance; in other words, participants who were more socially active had a better physical performance. Previous studies have likewise found a significant positive correlation between physical performance and social activity [41, 42]. One systematic review indicated that the greater the level of social activity, the greater the level of physical activity [42]. That review study also demonstrated a positive association between physical activity and social support among older adults who received social support from family members and friends. Higher levels of physical and social activity were, in turn, related to a lower incidence of depression and frailty among the older adults [42]. This connection between more social activity and lower depression may be explained by two phenomena: social activities are known to improve cognitive function and loneliness causes depression in older adults [43–46]. Meanwhile, in this study, social activity and frailty were moderately negatively correlated, meaning that those who had more social activity were less likely to be frail.

Finally, this study found a moderately negative correlation between physical performance and frailty that was affected by three mediating factors, namely, depression, well-being, and social activity. Next, we tested these mediating factors in the relationship between physical performance and frailty for parallel mediation.

#### 3.2.3. Parallel Mediation of the Relationship between Physical Performance and Frailty

On the relationship between physical performance and frailty, three mediation models were tested for each mediator (depression, well-being, and social) (Table 3), while parallel mediation (Figure 1) summarized the findings. The mediators of depression, well-being, and social activity acted as independent mediators in the relationship between physical performance and frailty in this study. Therefore, these findings might be used to supplement previous research on frailty and older adults in Taiwan. Our results confirmed that older adults with higher levels of depression tended to be more depressed than older people who were less depressed. Our study demonstrated that the effect of physical performance on frailty was partially mediated by depression among older adults. This finding is in line with previous studies that showed that older adults with more depression had a higher probability of suffering from frailty [14]. Moreover, depression is not only associated with frailty [13] but also it is affected by weak upper- and lower-body physical performance [34] and sedentary time [28, 32]. This may explain how depression mediates the effect of physical performance on frailty. In this study, older adults with impaired physical performance and a high level of depression had more severe frailty.

The results of this study also confirmed that older adults with higher levels of well-being were significantly less frail than older adults with lower levels of well-being. Previous studies have likewise found that well-being as a psychological state, which includes a sense of autonomy, happiness, and pleasure, is protective against the development of frailty [37]. However, physical performance is also relevant to positive mental well-being [35]. Moreover, cultural differences, such as differences in norms about emotions considered characteristic of mental health, may affect the measurement of well-being in older people [39]. Our findings indicate that well-being mediates the effect of physical performance on frailty. Not surprisingly, older adults with good physical performance and high levels of well-being were more robust in this study. In contrast, maladaptive psychological responses negatively impact psychological well-being and might contribute to frailty [39]. Therefore, health professionals should be aware that maintaining a strong sense of psychological well-being among older adults is important for their physical health.

Regarding the social domain in the older adult, living alone, social relation, social support, and social participation are related to frailty [47–49]. Moreover, a group of frailty experts agreed to include social activity in the development of an integral conceptual model of frailty [47]. Because it is important for an integrated human view, the social domain cannot be ignored in frailty assessment [48]. The social domains are related to (1) physical functioning, (2) ADL, (3) IADL, and (4) constraints on participation [50]. One study noted that the effects of social participation on cognitive function were greater in younger males than those in older males in Taiwan [43]. In sum, these studies have provided no evidence that social activity is a mediator of the relationship between physical performance and frailty. Therefore, the evidence related to the effect of social activity on physical performance and frailty is still at an early stage of understanding in this study.

## 4. Conclusion

To the best of our knowledge, the mediating factors in the relationship between physical performance and frailty have been discussed in several articles up to the present date. The limitations of the mediators of well-being are still unclear. This study provided preliminary cross-sectional support for a mediational model in which physical performance and frailty potentially explain the relationships among physical, psychological, and social factors among older adults in the community. To prevent frailty among older adult participants, comprehensive interventions should be designed for future research.

## Figures and Tables

**Table 1:** Cross-tabulation analysis between frailty and demographic characteristics of participants (*n* = 7,622).

Characteristics		Total	%	Health status		*p*value
				Robust (86.1%)	Frail (13.9%)	
Gender	Male	3,361	44.10	2,932	429	0.004
	Female	3,574	45.68	3,037	537	
	Missing	687	9.00			

Age	50–64	3,298	43.30	3,058	240	0.001
	65–85+	3,637	46.48	2,911	726	
	Missing	687	9.00			

Education	Lower	6,304	82.71	5,377	927	0.001
	Higher	630	8.29	591	39	
	Missing	688	9.00			

Marital status	Married	5,014	65.78	4,538	476	0.001
	Single	1,919	25.22	1,429	490	
	Missing	689	9.00			

Economic	Satisfaction	6,002	78.75	5,157	845	0.196
	Dissatisfaction	933	9.11	812	121	
	Missing	925	12.14			

Diseases in past year	No	3,408	44.71	3,160	248	0.001
	Yes	3,526	46.29	2,808	718	
	Missing	687	9.00			

Chronic diseases	None	6,698	87.78	5,772	926	0.001
	One kind	215	3.00	181	34	
	Two kinds	19	0.12	13	6	
	Three kinds	2	0.05	2	0	
	Four kinds	1	0.05	1	0	
	Missing	687	9.00			

**Table 2:** Descriptive statistics and Spearman's correlation analysis.

Variable	Mean	SD	1	2	3	4	5
(1) Physical performance	8.53	1.40	1.000				
(2) Depression	3.35	2.12	−0.245^∗∗^	1.000			
(3) Well-being	4.39	1.25	0.216^∗∗^	−0.225^∗∗^	1.000		
(4) Social activity	5.94	2.55	0.335^∗∗^	−0.221^∗∗^	0.204^∗∗^	1.000	
(5) Frailty	3.37	2.18	−0.562^∗∗^	0.376^∗∗^	−0.292^∗∗^	−0.444^∗∗^	1.000

^*∗*^ *p* < 0.05; ^∗∗^*p* < 0.01; ^∗∗∗^*p* < 0.001.

**Table 3:** Mediating models and the effects of depression, well-being, and social activity on the relationship between physical performance and frailty.

Model	Variables			Effect of *X* on *M*	Effect of *M* on *Y*	Direct effect	Indirect effect	Total effect
	*X*	*M*	*Y*	*A*	*B*	*c*′	(*a∗b*) 95% CI	*c*=*c*′ + *a∗b*
1	Physical performance	Depression	Frailty	−0.34 se = 0.02	0.20 se = 0.01	−1.03 se = 0.01	−0.05 (−0.05∼−0.04)	−1.10 se = 0.01
2		Well-being		0.22 se = 0.01	−0.19 se = 0.02	−1.03 se = 0.01	−0.03 (−0.03∼−0.02)	−1.07 se = 0.01
3		Social activity		0.57 se = 0.02	−0.18 se = 0.01	−1.03 se = 0.01	−0.07 (−0.07∼−0.06)	−1.13 se = 0.01

## Data Availability

The data that support the findings of this study are available from Health Data Science Center, Taiwan, but restrictions apply to the availability of these data, which were used under license for the current study, and so are not publicly available. The data are, however, available from the corresponding author upon reasonable request and with permission of the Taiwan Ministry of Health and Welfare.
